# Circulating CD14^bright^CD16^+^ ‘Intermediate’ Monocytes Exhibit Enhanced Parasite Pattern Recognition in Human Helminth Infection

**DOI:** 10.1371/journal.pntd.0002817

**Published:** 2014-04-24

**Authors:** Joseph D. Turner, Claire D. Bourke, Lynn Meurs, Moustapha Mbow, Tandakha Ndiaye Dièye, Souleymane Mboup, Katja Polman, Adrian P. Mountford

**Affiliations:** 1 Centre for Immunology and Infection, Department of Biology, University of York, York, United Kingdom; 2 Department of Biomedical Sciences, Institute of Tropical Medicine, Antwerp, Belgium; 3 Immunology Department of the Laboratory of Bacteriology and Virology of Aristide Le Dantec University Hospital, Dakar, Senegal; Centers for Disease Control and Prevention, United States of America

## Abstract

Circulating monocyte sub-sets have recently emerged as mediators of divergent immune functions during infectious disease but their role in helminth infection has not been investigated. In this study we evaluated whether ‘classical’ (CD14^bright^CD16^−^), ‘intermediate’ (CD14^bright^CD16^+^), and ‘non-classical’ (CD14^dim^CD16^+^) monocyte sub-sets from peripheral blood mononuclear cells varied in both abundance and ability to bind antigenic material amongst individuals living in a region of Northern Senegal which is co-endemic for *Schistosoma mansoni* and *S. haematobium*. Monocyte recognition of excretory/secretory (E/S) products released by skin-invasive cercariae, or eggs, of *S. mansoni* was assessed by flow cytometry and compared between *S. mansoni* mono-infected, *S. mansoni* and *S. haematobium* co-infected, and uninfected participants. Each of the three monocyte sub-sets in the different infection groups bound schistosome E/S material. However, ‘intermediate’ CD14^bright^CD16^+^ monocytes had a significantly enhanced ability to bind cercarial and egg E/S. Moreover, this elevation of ligand binding was particularly evident in co-infected participants. This is the first demonstration of modulated parasite pattern recognition in CD14^bright^CD16^+^ intermediate monocytes during helminth infection, which may have functional consequences for the ability of infected individuals to respond immunologically to infection.

## Author Summary

The parasite *Schistosoma* infects over 200 million people world-wide and can cause serious morbidity. Infection occurs following exposure to larvae (cercariae) which release excretory/secretory (E/S) material to aid their entry into exposed skin. Larvae mature into adult worms that produce hundreds of eggs per day which also release E/S material. Both sources of E/S material have the potential to stimulate the host’s innate immune system. Circulating monocytes are important cells that act as potential sentinels in the recognition of these E/S materials. Different sub-sets of human monocytes can be identified according to their expression of CD14 and CD16 but their role following infection with schistosome helminths has not been investigated. In the current study, three sub-sets (classical, intermediate and non-classical) were enumerated in individuals living in a region co-endemic for *S. mansoni* and *S. haematobium*. Although all three monocyte sub-sets bound to fluorescently-labelled schistosome E/S material, the intermediate sub-set had significantly enhanced ability to recognise cercarial and egg E/S in co-infected participants. This is the first demonstration that circulating human monocytes can recognize schistosome E/S antigens and that their ability to do so is modulated by infection which may affect the development of schistosome immunopathology and/or protective immunity.

## Introduction

Helminth parasites infect over 1 billion of the world’s population causing a range of overt morbid diseases and can exert substantial modulatory effects on the immune system [1], [2]. *Schistosoma mansoni* and *S. haematobium* are chronic, blood-dwelling, parasitic helminth infections of humans [3] and are co-endemic in many parts of Africa. Both species can cause life-threatening morbidities including damage to the intestines and liver (*S. mansoni*), or urogenital tract and kidneys (*S. haematobium*) [4].

Schistosome infection of the mammalian host is by skin penetration following exposure to waterborne cercariae [5], [6] which release excretory/secretory (E/S) material containing an abundance of glycosylated molecules [7] and proteases [8]. These E/S products aid penetration and migration of larvae, and consequently can act as initial stimuli of the cutaneous innate immune system [9]. Schistosome E/S products released in the first 3 hours after infection (0-3hRP) [10] stimulate both dendritic cells (DC) and macrophages (M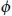) through binding of constituent ligands to pattern recognition receptors (PRR) such as Toll-like receptors (TLRs) [11], and C-type lectins (CLRs) including the mannose receptor (MR) [12]. These E/S products also have immune-modulatory effects on antigen presenting cells (APCs) such as DC *in vitro* and *in vivo* [13], particularly after repeated exposures, which can impact on downstream modulation of anti-schistosome responses and immunopathology in the liver [14].

Following migration and maturation, adult schistosome worms pair in the venous blood supplying the intestines (*S. mansoni*), or the bladder and urogenital tract (*S. haematobium*), and commence release of hundreds of eggs per day [15]. Mature eggs provide another source of glycosylated E/S products [16] termed egg secreted products (ESP). This E/S material may be critical in mediating the transit of eggs across host tissues to reach the external environment [17] and is implicated as a mediator of egg-related granulomatous immunopathology via induction of pro-fibrotic Th2 responses [18], [19]. Interestingly, an abundantly expressed ESP, Omega 1 [20], mediates Th2 priming via internalization into DC following ligation of the MR [21].

Despite the important role of E/S products in schistosome invasion, tissue migration and transmission of eggs, combined with their observed immunological priming and modulatory capacities in murine infection models, analysis of human immune responses to E/S material is very limited. Recently, we investigated cercarial E/S stimulation of whole blood cultures (WBC) from individuals from a region in Senegal which is co-endemic for *S. mansoni* and *S. haematobium* [22]. We identified significantly elevated levels of immune-regulatory IL-10, and increased ratios of IL-10:TNFα in infected individuals indicative of enhanced regulatory immune cell activity [22]. As the WBC culture supernatants were harvested at 24 hours post-stimulation with E/S material, the cytokines produced were most likely derived from the innate immune cell compartment (e.g. monocytes).

In this report, we extend our previous study by examining, for the first time in the context of human helminth infection, the parasite E/S pattern-recognition profiles of circulating monocyte sub-sets. We classified peripheral blood monocytes according to their expression of CD14 and CD16 in order to identify three sub-sets corresponding to ‘classical’ (CD14^bright^CD16^−^), ‘intermediate’ (CD14^bright^CD16^+^),and ‘non-classical’ (CD14^dim^CD16^+^) monocytes which have recently emerged as mediators of divergent immune functions during infectious disease [23]–[30]. Our study shows that intermediate CD14^bright^CD16^+^ monocytes have a greater ability to bind both cercarial and egg E/S products than other monocyte sub-sets, and that this capacity is greater in patients co-infected with both schistosome species compared to uninfected controls or those infected with *S. mansoni* only.

## Methods

### Ethics statement

This study was approved by the review board of the Institute of Tropical Medicine, Antwerp, the ethical committee of Antwerp University Hospital and ‘Le Comité National d’Ethique de la Recherche en Santé’ Dakar, Senegal. Written informed consent was obtained from all participants. All community members were offered a single dose of praziquantel (40 mg/kg) and mebendazole (500 mg) after the study to clear helminth infection.

### Study population and parasitology

Participants were recruited from the village of Diokhor Tack (N16.19°; W15.88°) in a region co-endemic for *S. haematobium* and *S. mansoni* [31]. Each participant provided two stool and two urine samples (with a minimum total volume of 10ml urine) on consecutive days to quantify schistosome eggs microscopically as described previously [22]. Participants were classified as ‘mono-infected’ if they had an *S. mansoni* egg count ≥1 egg in one or more of their stool samples and ‘co-infected’ if they were also found to have ≥1 *S. haematobium* egg in one or more of their urine samples. Participants infected with *S. haematobium* only were not included in this study. Of 54 participants who provided a blood sample, 4 were excluded for providing insufficient samples for parasitological analysis and 9 were excluded for providing insufficient blood volume to conduct all ligand binding assays.

### Innate immune ligands

The following ligands were used for binding studies of PBMCs: schistosome cercarial E/S (0-3hRP at 50 μg/ml), egg E/S product (ESP at 25 μg/ml), zymosan-coated AlexaFluor^488^ conjugated bio-particles (0.5×10^6^/tube; Life Technologies Ltd., Paisley, U.K.) as a positive control, and the fluorescein-labelled polyacrylamide glycoconjugate D-mannose (5 μg/ml; Lectinity Holding Inc., Moscow, Russia). Although zymosan is a yeast-derived ligand, zymosan bio-particles were selected as a positive control for the parasite E/S products because both ligands are heterogeneous in biochemical composition (containing carbohydrates, proteins and glycoproteins) and because like 0-3hRP, zymosan also stimulates in vitro cultured DCs to acquire a pro-Th2 activity [10]. D- mannose acted as a control for mannose receptor-mediated ligand binding, based on the knowledge that 0-3hRP contains an abundance of mannosylated glycans [7] which are important ligands for the macrophage MR [12]. 0-3hRP and ESP were prepared as previously described [10], [17], [32]. After isolation and purification, 0-3hRP and ESP were conjugated to AlexaFluor^488^ carboxylic acid 2,3,5,6-tetrafluorophenyl ester (Life Technologies Ltd, Paisley, U.K.) using established protocols [33].

### PBMC isolation and culture

Venous blood was collected in heparin coated-tubes [22], separated by density centrifugation (1400 rpm, 25 min, room temperature) on Ficoll (GE Healthcare, Pollards Wood, U.K.) and the resulting PBMC layer re-suspended at 10×10^6^ cells/ml in ice-cold phosphate buffered saline (PBS) containing 0.5% bovine serum albumin and 2 mM EDTA (Sigma Aldrich, St. Louis, U.S.A.). Aliquots of PBMC suspension (5×10^5^ cells) were transferred to 1.5 ml eppendorf tubes containing 50 μl of diluted ligands and monoclonal antibody (mAb) cocktail (see below) on ice. After a brief vortex, the suspension of PBMCs, ligands and mAbs was incubated on ice for 60 mins, with another vortex after 30 mins. For each participant, an aliquot of PBMC was incubated without ligands as a ligand-free control. PBMC were then washed with 900 μl ice cold PBS, pelleted at 800 *g* for 5 mins, before being re- suspended in 500 μl cell buffer containing 1% formaldehyde. PBMC were stored at 4°C in the dark before analysis by flow cytometry.

### Antibodies and cytometry

PBMC aliquots were surface-stained with fluorescently labeled anti-CD14 (conjugated to allophycocyanin; CD14-APC) and anti-CD16 (conjugated to eFluor^450^; CD16-ef450) mAb (eBioscience, San Diego, U.S.A.). Data was acquired with a Cyan flow cytometer (Beckman Coulter Ltd., High Wycombe, U.K.) and analysed using FlowJo software version 7.6.5 (TreeStar, Ashland, U.S.A.). Cells were gated according to forward- and side-scatter characteristics (SSC) and 5000–10000 total events acquired. An SSC^hi^ gate was used to select for cells with high granularity, which are primarily monocytes, whereas lymphocytes and NK cells were found in the gate for cells with low granularity. Polymorphonuclear granulocytes were mostly excluded by our Ficoll separation. SSC^hi^ PBMC were further sub-divided via CD14 and CD16 expression with gates determined relative to an aliquot of cells incubated with isotype control mAb to identify separate monocyte sub-sets. Ligand-free PBMC controls were used to determine threshold fluorescence intensity for AlexaFluor^488^ or fluorescein above which monocyte sub-sets were considered to be positive for ligand binding (ligand^+^ gate).

### Statistics

The software package IBM Statistics version 19 (Armonk, U.S.A.) was used for all statistical analyses. Mean proportions of monocyte sub-sets for each participant were calculated from proportions identified by cytometry of multiple aliquots of PBMC used for ligand binding assays (n  =  5/participant). Proportions of monocyte sub-sets within the SSC^hi^ gate, proportions of each sub-set in the SSC^hi^ ligand^+^ gate and proportions of ligand^+^ monocytes within each monocyte sub-set met the assumptions for parametric analysis. Thus, comparison between monocyte sub-sets was made using paired t-tests, and comparisons between schistosome infection groups were made using ANOVA. Post-hoc pair-wise comparisons were made for significant ANOVA using Fisher's test. As the total proportions of SSC^hi^ cells in the ligand^+^ gate and median fluorescence intensity (MFI) values did not meet the assumptions of parametric tests, even after transformation, statistical comparisons were made using non-parametric tests. The paired Wilcoxon test was used to compare the proportion of ligand^+^ SSC^hi^ cells relative to the ligand-free control. For comparison between infection groups, a Kruskal Wallis test was first used to determine statistical difference between groups and pair-wise Mann Whitney U tests were used post-hoc to identify which groups differed.

## Results

### Schistosome infection status does not affect the proportions of circulating monocyte sub-sets

The study population and schistosome infection status of the participants is detailed in Table 1 and comprised a total of 41 individuals aged 6 to 60 years old. When assigned by infection status, the three groups had similar sample sizes, age-range and sex ratios.

**Table 1:** Characteristics of the cohort by schistosome infection status.

	Un-infected	Infected	Co-infected
**n**	13	11	17
**Mean age (years) +/- SEM**	28.62 (4.67)	31.55 (5.69)	22.65 (3.73)
**Male: Female**	4∶9	3∶8	4∶13
**Geometric mean ** ***S. mansoni*** ** (eggs/g faeces) +/− SEM**	0	112.04 (235.63)	204.39 (255.32)
**Geometric mean ** ***S. haematobium*** ** (eggs/10 ml urine) +/− SEM**	0	0	11.94 (15.68)

**Figure 1:**
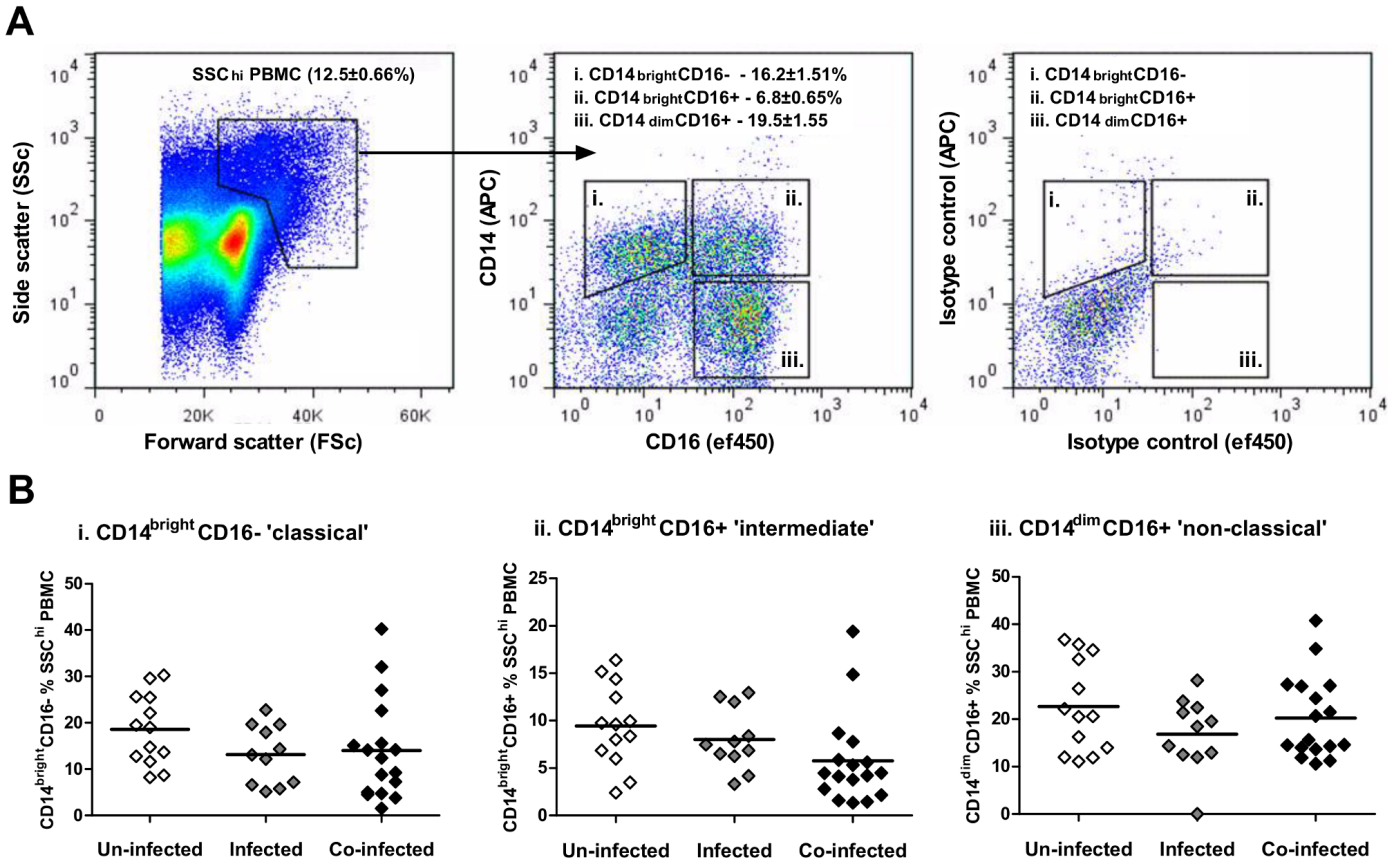
The proportions of SSC^hi^ monocyte sub-sets do not differ according to schistosome infection status. **A**). Representative flow plot showing distribution of total PBMC according to size and granularity, subsequently gated on cells with high granularity (i.e. SSC^hi^, left) and then analysed for their surface expression of CD14 and CD16 (centre) relative to cells from the same individual labelled with isotype control antibodies (right). Three discrete cell populations were identified (denoted by separate gates); i) CD14^bright^CD16^−^ ‘classical’ monocytes, ii) CD14^bright^CD16^+^ ‘intermediate’ monocytes, and iii) CD14^dim^CD16^+^ ‘non-classical’/‘inflammatory’ monocytes. Numerical values indicate mean proportions of each sub-set within the SSC^hi^ gate ± standard error of the mean. **B**). The proportions of each monocyte sub-set within the SSC^hi^ gate (defined above) in all study participants plotted according to schistosome infection status (n = 41). Data points are for individual participants in each infection group, with horizontal bars representing the mean value. ANOVA showed no significant differences in each monocyte sub-set between the three infection groups (p>0.05).

Following labelling with mAbs specific to CD14 and CD16, three discrete populations of SSC^hi^ monocytes were identified according to their recent characterisation and nomenclature in human peripheral blood; i) CD14^bright^CD16^−^ ‘classical’ monocytes, ii) CD14^bright^CD16^+^ ‘intermediate’ monocytes and iii) CD14^dim^CD16^+^ ‘non-classical’ monocytes (Fig. 1A). Of the two CD14^+^ monocyte sub-sets, the CD14^bright^CD16^−^ population was more abundant (16.2±1.5% of total SSC^hi^) than the CD14^bright^CD16^+^ (6.8±0.7% of total SSC^hi^) sub-set. Although the CD14^dim^CD16^+^ population was the most abundant overall (19.5±1.6% of total SSC^hi^), it may include a small number of CD14^−^ granulocytes as previously noted [28] but CD14^dim/-^CD16^+^ NK cells were excluded from our analysis as they do not have a high granularity phenotype defined as SSC^hi^.

When the relative abundances of the three monocyte sub-sets were compared by infection status, no statistically significant differences in the proportions of each sub-set between the un-infected, infected with *S. mansoni* only, or co-infected with *S. mansoni* and *S. haematobium* groups were identified (Fig. 1B, p>0.05 for all comparisons).

### Monocyte sub-sets differ in their capacity to bind schistosome E/S antigens

**Figure 2:**
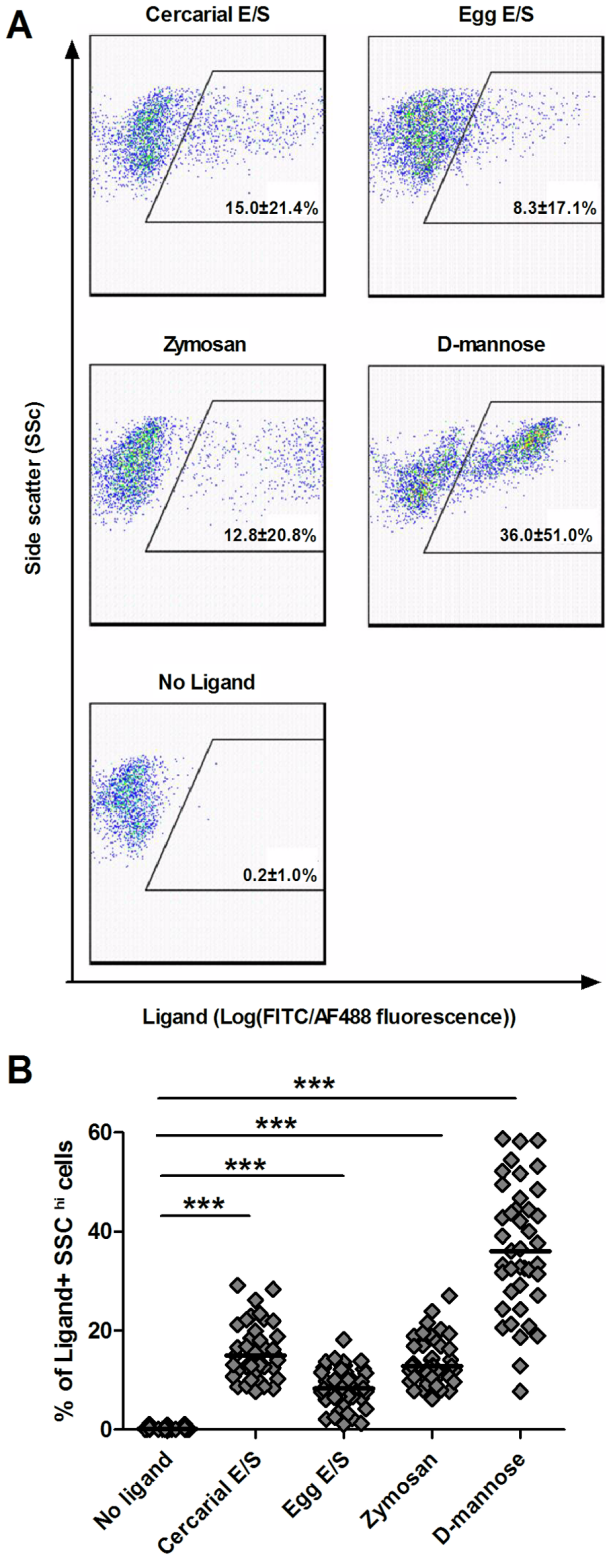
SSC^hi^ monocytes bind cercarial E/S and egg E/S material. **A**). Representative flow cytometry dot plots showing binding of Alexafluor^488^-conjugated cercarial E/S products (0-3hRP), Alexafluor^488^-conjugated egg E/S products (ESP), Alexafluor^488^-conjugated zymosan-coated bio-particles, and Fluorescein-conjugated D-mannose, to SSC^hi^ monocytes following incubation for 60 mins. Populations of ligand^+^ cells were identified for each ligand via a ligand^+^ gate set relative to cells incubated without antigen (No ligand control). Plots of ligand binding are representative of data accrued from all study participants. Numerical values are the median for binding of each ligand ± the range. **B**). Data shows the proportions of SSC^hi^ cells in the ligand^+^ gate for each participant relative to cells from the same individuals incubated without ligands (horizontal bars indicate the median value; Paired Wilcoxon test, ***p<0.001, ligand^+^ *versus* no ligand control, n = 41).

The ability of SSC^hi^ cells to recognise schistosome and non-schistosome pathogen-associated molecular patterns (PAMPs) was investigated by examining their ability to bind each of the fluorescently-conjugated ligands (Fig. 2A). SSC^hi^ monocytes bound the two schistosome E/S products, zymosan bio-particles and D-mannose and had significantly greater proportions of cells within the ligand^+^ gate than their corresponding ligand-free controls (Fig. 2B, p<0.001 for all comparisons).

**Figure 3:**
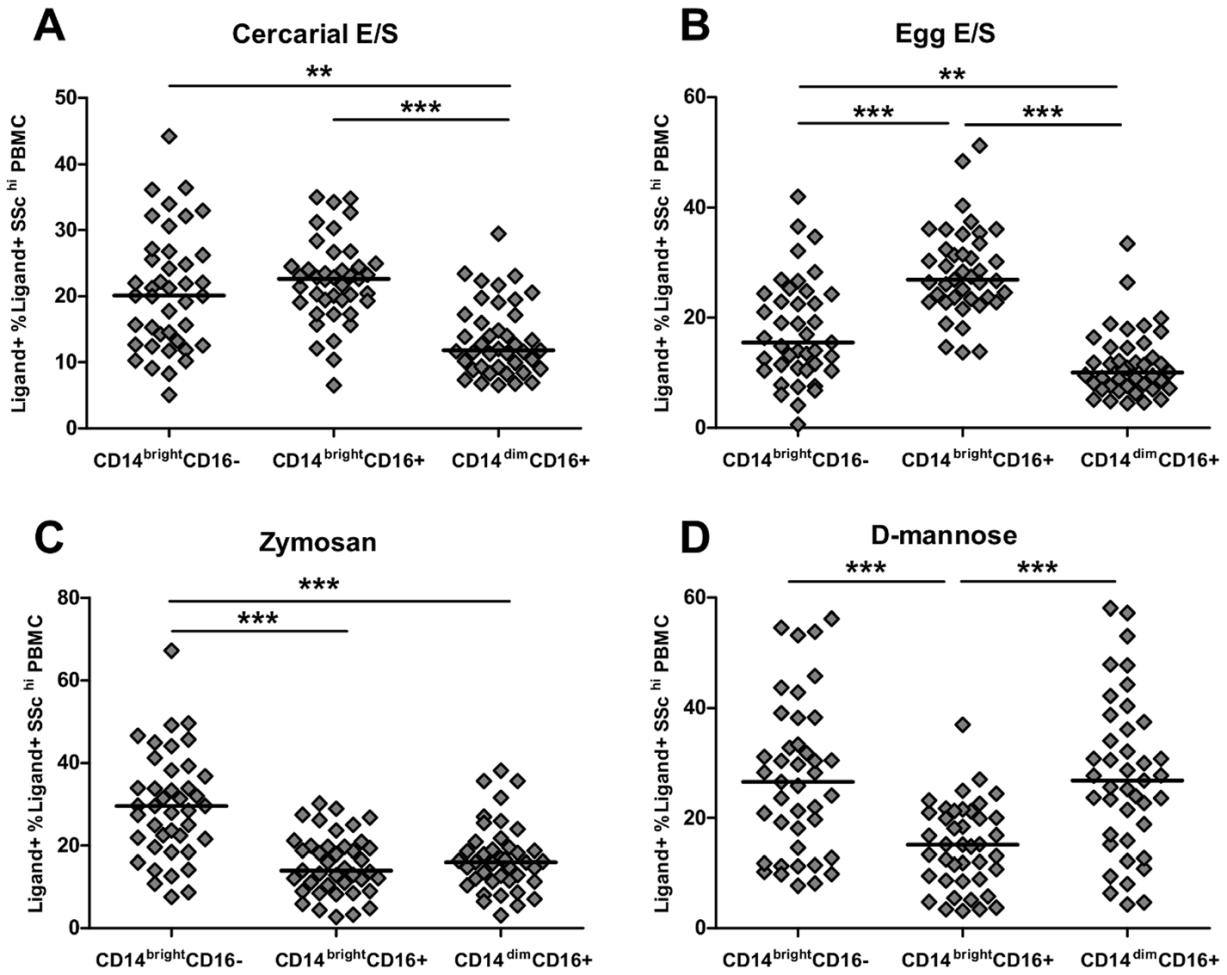
CD14^bright^CD16^+^ intermediate monocytes preferentially bind cercarial and egg E/S but not Zymosan or D-mannose. Proportions of ligand^+^ SSC^hi^ PBMC subdivided into monocyte sub-sets according to their expression of CD14 and CD16. Binding of **A**) Alexafluor^488^-conjugated cercarial E/S products, **B**) Alexafluor^488^-conjugated egg E/S products, **C**) Alexafluor^488^-conjugated zymosan-coated bio-particles, and **D**) Fluorescein-conjugated D-mannose, to different monocyte sub-sets following incubation for 60 mins. Populations of ligand^+^ cells were identified for each ligand relative to cells incubated without antigen (Fig.2A). Data was accrued from all study participants (n = 41; horizontal bars indicating the median value; Paired Wilcoxon test, ***p<0.001, **p<0.01).

Within the SSC^hi^ population, CD14^bright^ monocytes (both the classical CD16^−^ and intermediate CD16^+^ sub-sets) were more efficient at binding to cercarial E/S than the CD14^dim^ non-classical population (Fig. 3A; CD14^bright^CD16^−^ t: 3.57, p<0.01, CD14^bright^CD16^+^ t: 6.30, p<0.001). Intermediate CD14^bright^CD16^+^ monocytes were also the most efficient at binding egg E/S products compared to the other monocyte sub-sets (Fig. 3B; *cf.* CD14^bright^CD16^−^ t: 6.438, p<0.001, and *cf.* CD14^dim^CD16^+^ t: 9.29, p<0.001). However, CD14^bright^CD16^+^ intermediate monocytes were less efficient in their binding of the control ligands zymosan and D-mannose than the other monocyte sub-sets (Fig. 3C & D). A greater proportion of classical CD14^bright^CD16^−^ monocytes bound egg E/S than non-classical CD14^dim^CD16^+^monocytes (Fig. 3B; t: 2.87, p = 0.007) and classical monocytes also bound zymosan with the greatest efficiency compared to intermediate (Fig. 3C; t: 6.41, p<0.001) and non-classical monocytes (t: 4.40, p<0.001). The classical and non-classical sub-sets had equivalent binding efficiency to D-mannose (Fig 3D; t: 0.09, p = 0.931) and both bound D-mannose with greater efficiency than the intermediate subset (CD14^bright^CD16^−^ t: 5.45, p<0.001, CD14^dim^CD16^+^ t: 4.48, p<0.001).

### Schistosome infection leads to increased schistosome E/S antigen binding by intermediate monocytes

**Figure 4:**
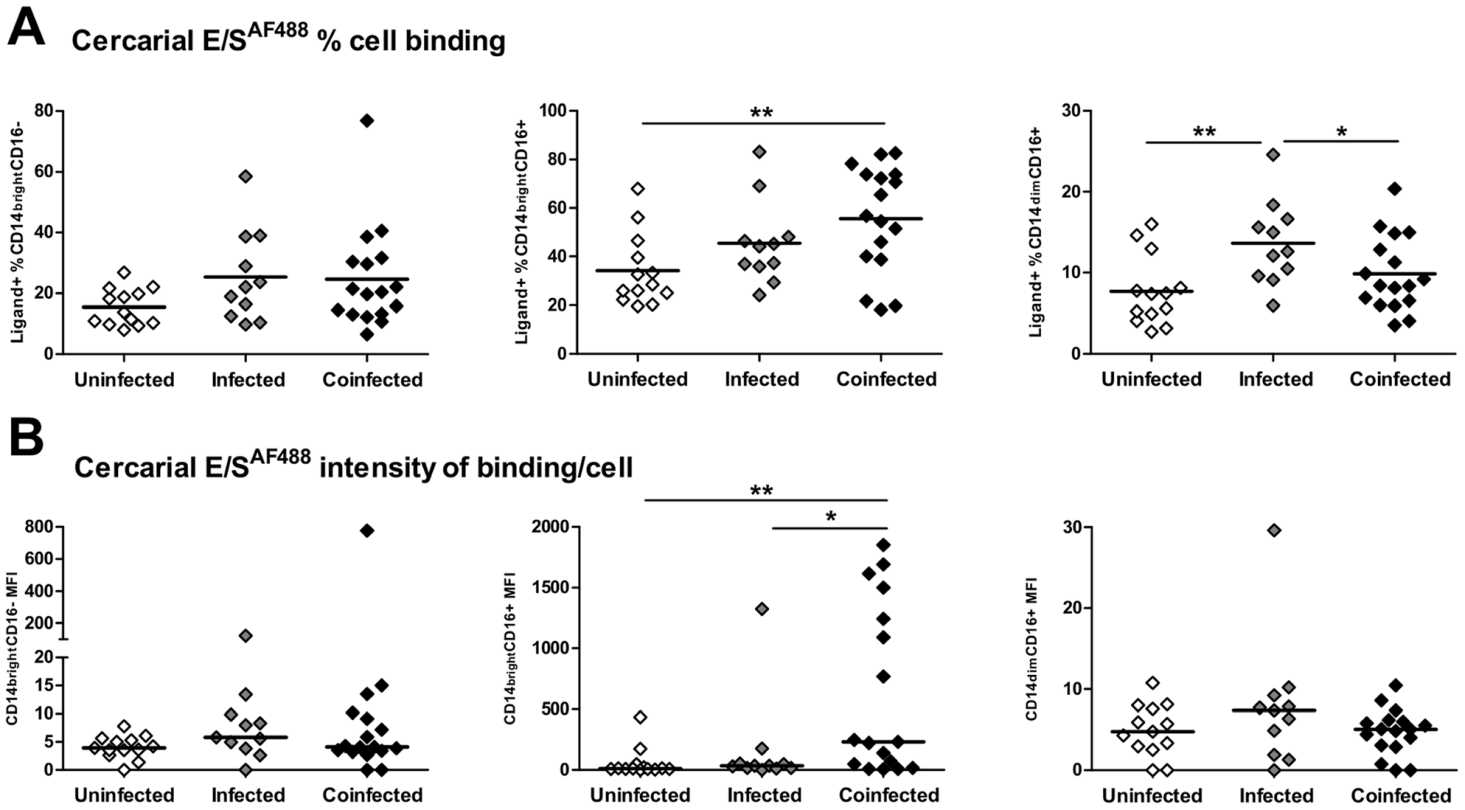
CD14^bright^CD16^+^ intermediate monocytes from co-infected individuals bind cercarial E/S ligands more efficiently than those from un-infected individuals. **A**). The proportions of each monocyte sub-set that bound cercarial E/S compared between participants grouped according to schistosome infection status (un-infected, infected and co-infected). Horizontal bars denote mean proportions of ligand^+^ cells for each group. Post-hoc pairwise comparisons (Fisher's least significant difference tests) are shown where ANOVA was significant, *p<0.05, **p<0.01. **B**). MFI for each sub-set incubated with cercarial E/S illustrating the relative quantity of ligand binding. Bars denote median MFI for each infection group. Post-hoc pairwise Mann Whitney U comparisons are shown where non-parametric Kruskal Wallis tests were significant, *p<0.05, **p<0.01. Cell proportions in the ligand^+^ gate and MFI for cells incubated without ligand were subtracted from those of cells incubated with fluorescently-labelled cercarial E/S products prior to comparison between infection groups.

Having established functional distinctions between the three SSC^hi^ populations in their ligand binding capacity, we investigated whether ligand binding within each monocyte sub-set depended upon participant infection status within the study population. Similar proportions of classical monocytes bound cercarial E/S in all 3 infection groups (Fig. 4A; F_2, 38_: 2.12, p = 0.135). In contrast, ligand uptake by intermediate monocyte was influenced by infection status (F_2, 38_: 4.93, p = 0.013) with a significantly greater proportion of intermediate monocytes from co-infected participants binding to cercarial E/S than those from uninfected subjects (Fig. 4A, mean: 55.64±5.31% *versus* 34.10±4.08%, *p* = 0.003). A greater proportion of non-classical monocytes also bound cercarial E/S products in mono-infected compared with un-infected (Fig. 4A, 13.64±1.55% *versus* 7.69±1.19%, *p* = 0.004) or co-infected participants (9.83±1.12%, *p* = 0.041).

In terms of the quantity of cercarial E/S bound by each monocyte sub-set (determined by MFI), although classical monocytes bound similar amounts in all infection groups (Fig. 4B, Kruskal Wallis; *Χ* ^2^: 2.90, *p* = 0.235), the amount bound by intermediate monocytes differed according to participant infection status (Fig. 4B, Kruskal Wallis; *Χ* ^2^: 10.01, *p* = 0.007). Hence, CD14^bright^CD16^+^ intermediate monocytes from co-infected patients bound significantly greater quantities of antigen as judged by their higher MFI ( = 231.90) than either mono-infected ( = 34.34, *p*<0.05) or uninfected participants ( = 10.70, *p*<0.01). In fact, intermediate monocytes from some co-infected individuals were particularly efficient at binding cercarial E/S (i.e. 7 co-infected participants bound >2-fold greater quantities of cercarial E/S than the group median, Fig. 4B). There was no significant difference between the three infection groups in the amount of cercarial E/S bound by non-classical monocytes (Fig. 4B, Kruskal Wallis; *Χ* ^2^: 1.89, p = 0.388).

**Figure 5:**
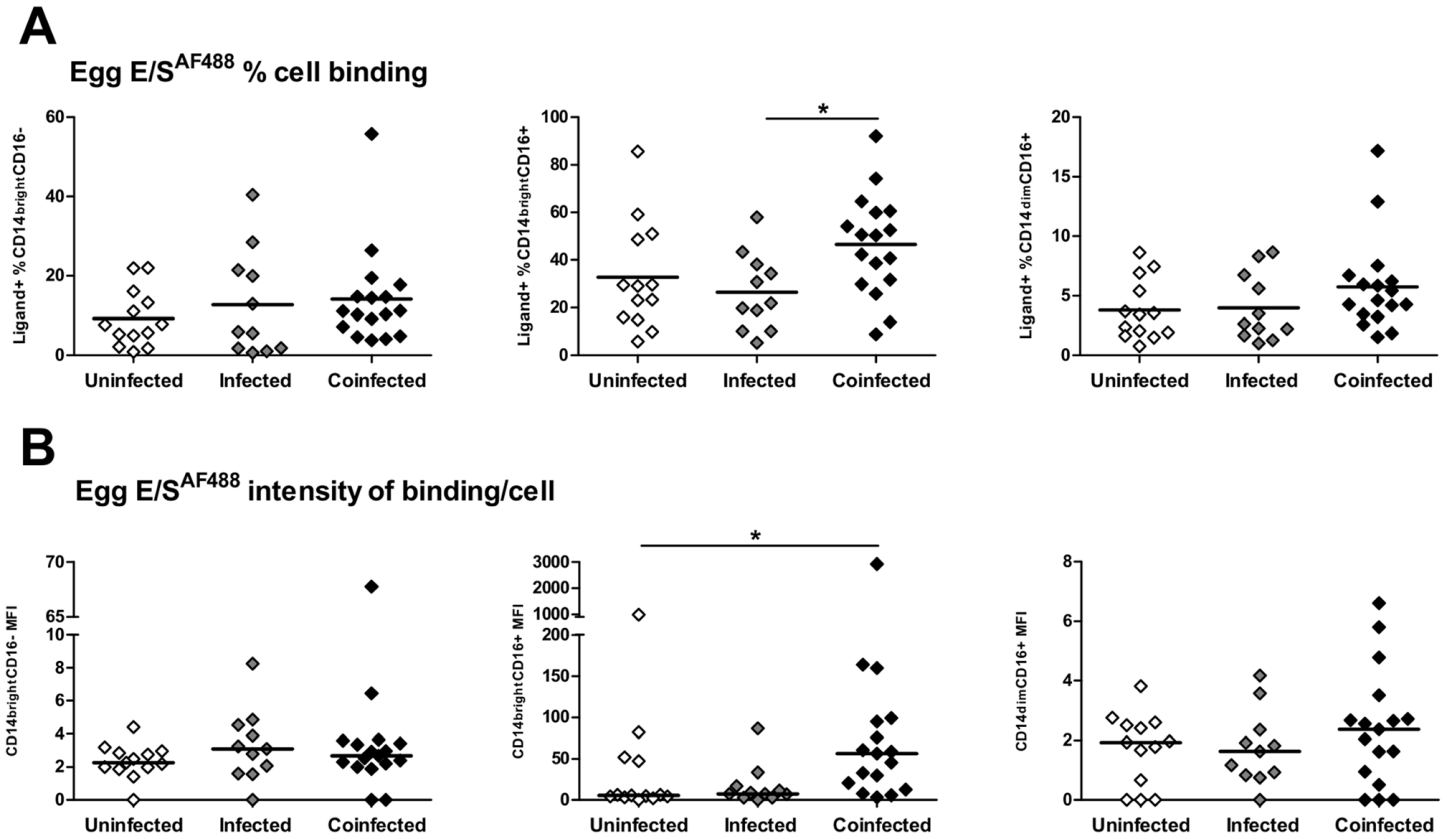
CD14^bright^CD16^+^ intermediate monocytes from co-infected individuals bind egg E/S ligands more efficiently than those from un-infected individuals. **A**). The proportions of each monocyte sub-set that bound Alexafluor^488^-conjugated egg E/S compared between participants grouped according to schistosome infection status (un-infected, infected and co-infected). Horizontal bars denote mean proportions of ligand^+^ cells for each group. Post-hoc pairwise comparisons (Fisher's least significant difference tests) are shown where ANOVA was significant, *p<0.05, **p<0.01. **B**). MFI for each monocyte sub-set incubated with cercarial E/S illustrating the relative quantity of antigen binding compared by infection status. Bars denote median MFI for each infection group. Post-hoc pairwise Mann Whitney U comparisons are shown where non-parametric Kruskal Wallis tests were significant, *p<0.05, **p<0.01. Cell proportions in the ligand^+^ gate and MFI for cells incubated without ligand were subtracted from those of cells incubated with fluorescently-labelled egg E/S products prior to comparison between infection groups.

The proportions of classical monocytes that bound schistosome egg E/S products were similar between the three infection groups (Fig. 5A, F_2, 38_: 0.693, p = 0.506), as were the amounts of egg E/S bound by this sub-set (Fig. 5B, Kruskal Wallis; *Χ* ^2^: 2.11, *p* = 0.348). However, significant infection-related differences were evident in the intermediate monocyte population (Fig. 5A; F_2, 38_: 3.59, *p* = 0.037). Greater proportions of intermediate monocytes from co-infected subjects bound egg E/S (Fig. 5A; 46.53±5.14%) than those isolated from mono-infected participants (12.72±4.00%; *p* = 0.015). There was also a non-significant trend for a greater proportion of intermediate monocytes recognising egg E/S in co-infected *versus* un-infected patients (Fig. 5A; 32.72±6.28%, *p* = 0.075). In addition, intermediate monocytes varied in the amount of bound egg E/S according to infection status (Fig. 5B; Kruskal Wallis; *Χ* ^2^: 9.60, *p* = 0.008) with those from the co-infected group binding significantly greater quantities (MFI: 56.03) than those from un-infected individuals (Fig. 5B; MFI: 5.30, *p* = 0.015). There was no difference in the proportions of egg E/S^+^ non-classical monocytes (Fig.5A, F_2, 38_: 1.64, p = 0.208), nor in the amount of egg E/S uptake by this sub-set between the 3 infection groups (Fig. 5B, Kruskal Wallis; *Χ* ^2^: 0.972, *p* = 0.615).

Evaluation of the binding recognition profiles for zymosan (a yeast- derived bioparticle) and D-mannose (a purified glycan) by the different monocyte sub-sets between schistosome infection groups did not reveal any significant differences (Supplementary Figs. S1 & S2). Although not directly comparable with our schistosome-derived E/S products, these observations confirm that the significantly elevated proportions of CD14^bright^CD16^+^ intermediate monocytes from co-infected participants that bound cercarial, or egg E/S products were not due to an increase in non-specific binding regardless of the ligand.

## Discussion

Monocytes are recruited to tissue sites of inflammation and infection and are precursors of specific M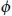 and DC populations at tissue sites (e.g. the skin and intestines). Thus, monocytes potentially have both innate immune and subsequent APC functions in response to different stages of the schistosome parasite in multiple tissue sites. Although human circulating monocytes have traditionally been identified on the basis of CD14 (a lipopolysaccharide co-receptor) expression, more recently they have been further subdivided according to the expression of the low affinity Fc receptor, CD16, which could define their binding to various ligands and their immune function [28]. In mice, which are an established in vivo experimental model of human schistosomiasis, monocytes have been defined on the basis of expression of the surface markers Ly6C, CD115 and the chemokine receptor CCR2, and are thought to be selectively recruited to inflamed tissues [34]. However, monocytes that are Ly6C^-^ are thought to differentiate into alternatively activated M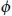 [34], [35] which dominate after multiple exposures to schistosome cercariae and following chronic long-term infection [14], [36]. However, due to limited characterization of the distinctions between murine and human monocyte markers, it is difficult to directly translate findings with murine models into human disease. On the other hand, a consensus on the definition of monocyte sub-sets in humans based upon their pattern of labeling with anti-CD14 and anti-CD16 mAbs is emerging, despite ongoing debate over the functional distinctions between these sub-sets (see commentary in [29]). Therefore, in our study of PBMC monocytes collected from individuals inhabiting a schistosome-endemic region of northern Senegal, SSC^hi^ monocyte sub-sets were classified as being CD14^bright^CD16^-^ (‘classical’), CD14^bright^CD16^+^ (‘intermediate’), and CD14^dim^CD16^+^ (‘inflammatory’/‘non-classical’).

We are not aware of a precedent study that has examined the ability of human monocyte sub-sets to differentially bind innate immune cell ligands and thus our study is the first to demonstrate functional distinctions between the three monocyte sub-sets in their capacity for pattern recognition of schistosome-derived E/S. In particular, we show that CD14^bright^ monocytes are more efficient at binding to schistosome E/S antigens than CD14^dim^ non-classical monocytes, highlighting a potential role for classical and intermediate monocytes in innate sensing of human schistosome infections. Moreover, our study is the first to demonstrate that schistosome infection status affects the binding of CD14^bright^CD16^+^ intermediate monocytes, but not the other two monocyte sub-sets, to schistosome E/S ligands. We show that a significantly greater proportion of CD14^bright^CD16^+^ intermediate monocytes from *S. mansoni* and *S. haematobium* co-infected participants recognize and bind schistosome E/S products from cercariae and mature eggs, than the same sub-set of monocytes obtained from mono- and uninfected subjects. Furthermore, this intermediate sub-set also binds greater quantities of E/S antigen. This may be due to greater numbers of *S. mansoni* eggs in co-infected compared to mono-infected patients (Table 1), to the additional presence of *S. haematobium*, or to a combination of both factors. Together, this data indicates that schistosome infection affects the surface receptor repertoire of CD14^bright^CD16^+^ monocytes, enabling them to become more sensitive to recognition of schistosome-secreted molecules, which may enhance subsequent recruitment of this sub-set to tissue sites of infection. This is potentially of significance in the development of schistosome-specific protective immunity or immunopathology. Indeed, a functional role for intermediate monocytes in the development of severe malaria has previously been proposed [37].

Candidate monocyte surface receptors that may be influenced by infection status and are known to be involved in pattern recognition of schistosome cercarial and egg E/S ligands include surface TLRs (2 and 4) and the phagocytic C-type lectin, MR, previously implicated in glycosylated schistosome molecule recognition [11], [12], [21], [38]-[40]. Ligation of MR by cercarial E/S has an immune modulatory effect [12], possibly acting on TLR signaling [41] as proposed for other schistosome-derived glycans [39]. However, because D-mannose was not differentially recognized in the three infection groups in our study, schistosome E/S recognition in intermediate monocytes may be independent of infection-related changes in MR expression. Furthermore, as binding of zymosan (purified yeast cell wall); a commonly encountered PAMP recognized by both TLR2 and the β-glucan C-type lectin, Dectin-1 [42], to each of the three monocyte sub-sets did not vary significantly between infection groups, it is unlikely that increased recognition of parasite E/S in infected individuals cause alterations in the TLR2 and Dectin1 PRR complex.

The differential expression of surface receptors, other than CD14 and CD16, such as TLRs and C-type lectins, was beyond the scope of this first investigation of monocyte heterogeneity in helminth-infected humans. However, our findings indicate that determining which PRRs are differentially expressed between the various sub-sets in the context of infection status would be pertinent. Moreover, since it is unknown at present whether differential PRR expression is dependent upon the origin (e.g. bacterial, fungal, protozoan or helminth) of the stimulatory ligands to which they are exposed, an investigation of PRR expression by different monocyte sub-sets in response to schistosome antigens alongside appropriate defined control antigens from other pathogen sources (e.g. zymosan), is a valid area for further investigation. In addition, it would be desirable to determine whether binding of the different parasite E/S products to these PRRs proceeds to endocytosis and how this impacts on monocyte-derived APC function. Previous investigations have already begun to further subdivide the three monocyte sub-sets described here according to surface expression of Major histocompatibility molecules and chemokine receptors [37], [43] although their functional relevance to pattern recognition by monocytes has yet to be investigated. It would also be instructive to determine whether activation, or regulatory signals, such as secretion of different cytokines and chemokines, are induced in the respective monocyte sub-sets following ligation of parasite E/S products at the cell surface.

Monocytes expressing CD16 have been regarded as ‘pro-inflammatory’ according to their cytokine secretion profile (i.e. high TNFα and low IL-10 in response to LPS) and their ability to present antigen, suggesting they are more mature than the CD16^−^ classical monocyte sub-set [24]. CD16^+^ monocytes are also more liable to develop into M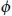 or DC [44] and the CD14^bright^CD16^+^ intermediate sub-set is usually expanded under inflammatory disorders [28], [45], although our data indicates that this is not the case for schistosomiasis. However, the intermediate monocyte sub-set has also been identified as a major source of the regulatory cytokine IL-10 [46]. The latter contention is supported by data suggesting that CD14^bright^CD16^+^ intermediate monocytes may act in an anti-inflammatory manner in response to infectious pathogens such as *Plasmodium* protozoa [37]. This raises the question as to whether the function of CD14^bright^CD16^+^ intermediate monocytes (i.e. having a pro-inflammatory *versus* a regulatory role) depends on the specific molecular composition of the stimulatory microbial or inflammatory ligand. Interestingly, it has recently been reported that CD16^+^ monocytes, which are abundant (∼40%) in patients infected with *Mycobacterium tuberculosis*, fail to differentiate into mature DC [47] and can adversely affect the ability of classical CD14^+^ monocytes to differentiate into DC [47]. Therefore, CD16^+^ monocytes under the influence of specific microbial ligands may give rise to immune-regulatory DC, or divert differentiation of tissue macrophages to having anti-inflammatory properties [48]. In this context, we have previously shown that murine bone-marrow derived DC exposed to cercarial E/S fail to mature taking on a ‘modulated’ or ‘regulatory’ phenotype [32] and release abundant regulatory IL-10 [10]. Thus, in light of the report that CD14^bright^CD16^+^ intermediate monocytes produce abundant IL-10 [46], it would be pertinent to determine whether the elevated levels of IL-10 released by WBC from schistosome-infected patients in response to stimulation with cercarial E/S [22] are due to elevated numbers of CD14^bright^CD16^+^intermediate monocytes that have bound to cercarial and/or egg E/S products.

In conclusion, our study shows that circulating CD14^bright^CD16^+^ intermediate monocytes have a hitherto un-appreciated potential to specifically bind schistosome E/S material which may ultimately shape the development and function of monocyte-derived myeloid cells (e.g. M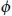 and DC) recruited to parasitized tissue sites during schistosome infection. In addition, the ability of CD14^bright^CD16^+^ intermediate monocytes to recognize parasite-derived E/S molecules is enhanced in schistosome-infected patients compared with uninfected individuals, suggesting a mechanism of modulation in surface-expression of parasite pattern recognition receptors on this specific monocyte sub-set. Future lines of study should include: identification of the innate immune cell PRRs involved in the binding of E/S products, investigation of the cytokine secretion and activation profile of ligand^+^ monocytes, the fate of bound ligands (e.g. internalization and intracellular processing), and analysis of the functional potential of E/S-exposed monocytes (e.g. phagocytosis and/or antigen presentation). In spite of these unknowns, our study indicates that exposure to schistosome-derived E/S products may profoundly influence the function of circulating monocyte sub-sets, which in turn may have substantial modulating effects on human immune reactivity. Importantly, differences in the responsiveness of circulating APC precursors to schistosome E/S material may impact upon permissiveness to invading cercariae at the cutaneous site of infection and the development of immunopathology around eggs sequestered in host tissues.

## Supporting Information

Figure S1 **The efficiency of the three monocyte sub-sets to bind zymosan does not differ according to infection status.** **A**). The proportions of each monocyte sub-set that bound Alexafluor^488^-conjugated zymosan bio-particles compared between participants grouped according to schistosome infection status (un-infected, infected and co-infected). Horizontal bars denote mean proportions of ligand^+^ cells for each group. Post-hoc pairwise comparisons (Fisher's least significant difference tests) are shown where ANOVA was significant, *p<0.05, **p<0.01. **B**). MFI for each monocyte sub-set incubated with zymosan bio-particles illustrating the relative quantity of antigen binding compared by infection status. Bars denote median MFI for each infection group. Post-hoc pairwise Mann Whitney U comparisons are shown where non-parametric Kruskal Wallis tests were significant, *p<0.05, **p<0.01. Cell proportions in the ligand^+^ gate and MFI for cells incubated without ligand were subtracted from those of cells incubated with fluorescently-labelled ligands prior to comparison between infection groups.
(TIF)

Figure S2 **The efficiency of the three monocyte sub-sets to bind D-mannose does not differ according to infection status.** **A**). The proportions of each monocyte sub-set that bound fluorescein-conjugated D-mannose compared between participants grouped according to schistosome infection status (un-infected, infected and co-infected). Horizontal bars denote mean proportions of ligand^+^ cells for each group. Post-hoc pairwise comparisons (Fisher's least significant difference tests) are shown where ANOVA was significant, *p<0.05, **p<0.01. **B**). MFI for each monocyte sub-set incubated with D-mannose, illustrating the relative quantity of antigen binding compared by infection status. Bars denote median MFI for each infection group. Post-hoc pairwise Mann Whitney U comparisons are shown where non-parametric Kruskal Wallis tests were significant, *p<0.05, **p<0.01. Cell counts in the ligand^+^ gate and MFI for cells incubated without ligand were subtracted from those of cells incubated with fluorescently-labelled ligands prior to comparison between infection groups.
(TIF)
