# Supplementary figures and images for: Circulating CD14brightCD16+ ‘Intermediate’ Monocytes Exhibit Enhanced Parasite Pattern Recognition in Human Helminth Infection
Source: PLoS Negl Trop Dis. 2014 Apr 24;8(4):e2817. doi: 10.1371/journal.pntd.0002817 (PMC3998941; doi:10.1371/journal.pntd.0002817)

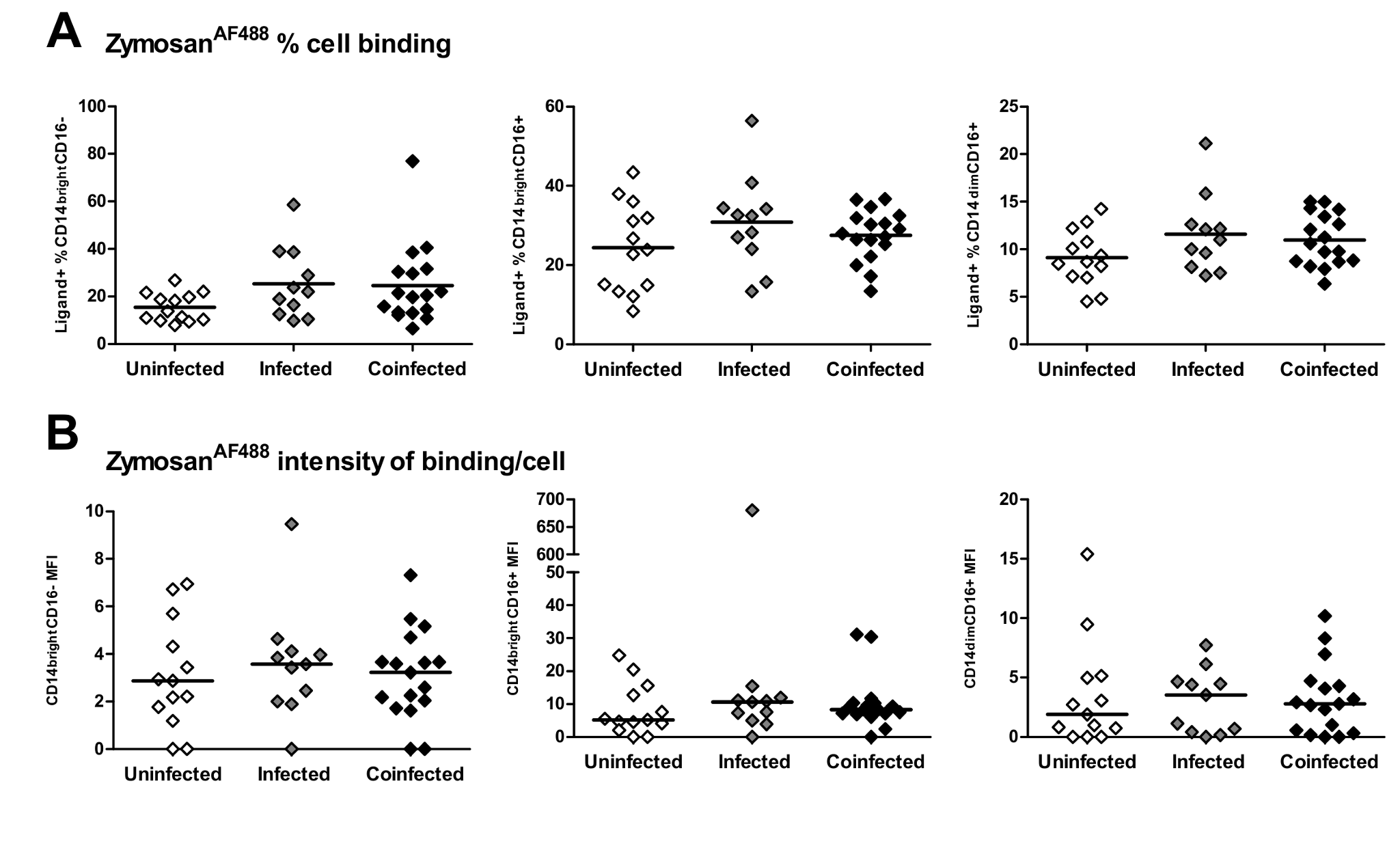

Supplement: Figure S1 — The efficiency of the three monocyte sub-sets to bind zymosan does not differ according to infection status. A). The proportions of each monocyte sub-set that bound Alexafluor488-conjugated zymosan bio-particles compared between participants grouped according to schistosome infection status (un-infected, infected and co-infected). Horizontal bars denote mean proportions of ligand+ cells for each group. Post-hoc pairwise comparisons (Fisher's least significant difference tests) are shown where ANOVA was significant, *p<0.05, **p<0.01. B). MFI for each monocyte sub-set incubated with zymosan bio-particles illustrating the relative quantity of antigen binding compared by infection status. Bars denote median MFI for each infection group. Post-hoc pairwise Mann Whitney U comparisons are shown where non-parametric Kruskal Wallis tests were significant, *p<0.05, **p<0.01. Cell proportions in the ligand+ gate and MFI for cells incubated without ligand were subtracted from those of cells incubated with fluorescently-labelled ligands prior to comparison between infection groups. (TIF) [file pntd.0002817.s001.tif]

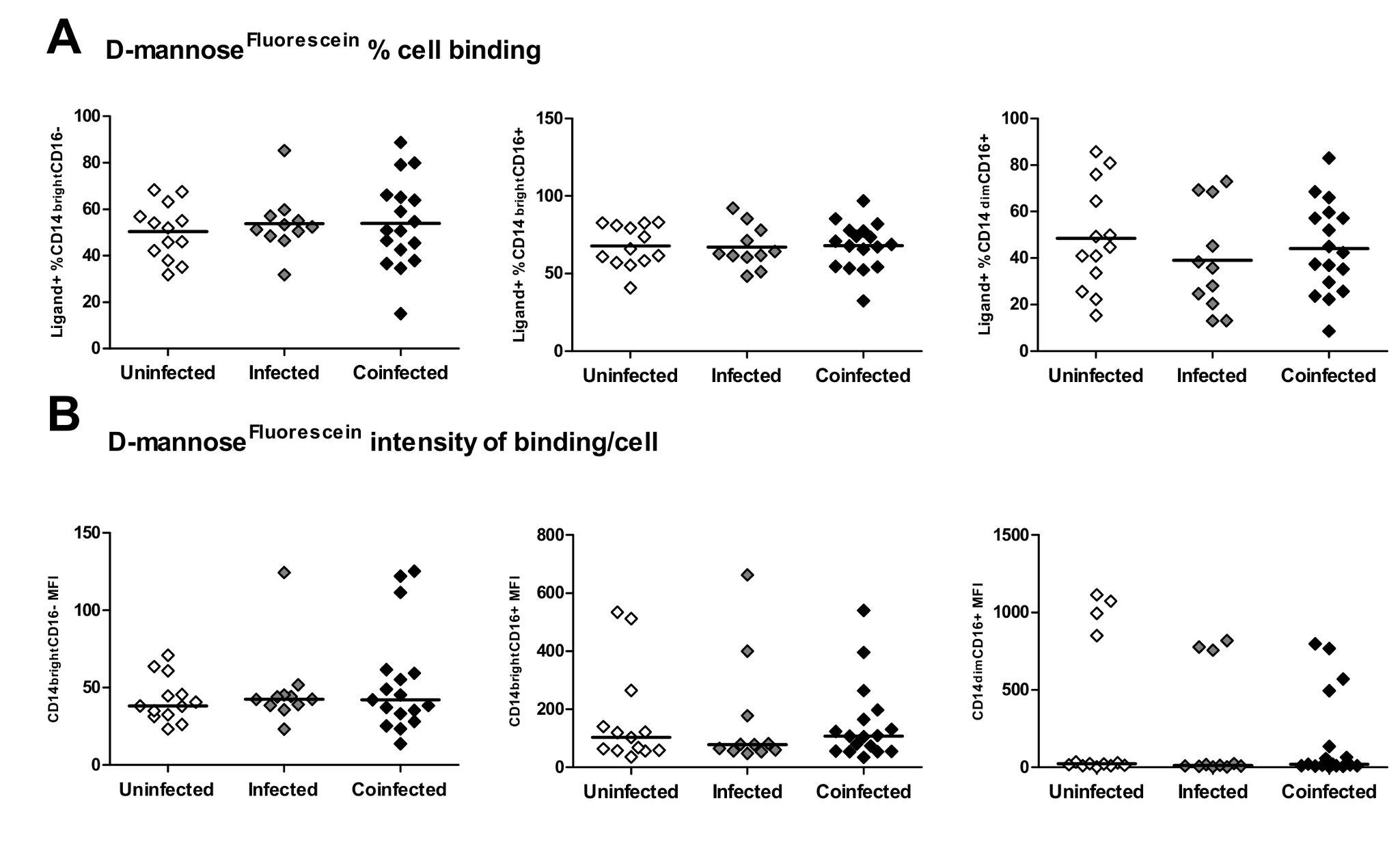

Supplement: Figure S2 — The efficiency of the three monocyte sub-sets to bind D-mannose does not differ according to infection status. A). The proportions of each monocyte sub-set that bound fluorescein-conjugated D-mannose compared between participants grouped according to schistosome infection status (un-infected, infected and co-infected). Horizontal bars denote mean proportions of ligand+ cells for each group. Post-hoc pairwise comparisons (Fisher's least significant difference tests) are shown where ANOVA was significant, *p<0.05, **p<0.01. B). MFI for each monocyte sub-set incubated with D-mannose, illustrating the relative quantity of antigen binding compared by infection status. Bars denote median MFI for each infection group. Post-hoc pairwise Mann Whitney U comparisons are shown where non-parametric Kruskal Wallis tests were significant, *p<0.05, **p<0.01. Cell counts in the ligand+ gate and MFI for cells incubated without ligand were subtracted from those of cells incubated with fluorescently-labelled ligands prior to comparison between infection groups. (TIF) [file pntd.0002817.s002.tif]
